# Do antipsychotic medications work: An exploration using competency to stand trial as the functional outcome

**DOI:** 10.1017/S1092852924002372

**Published:** 2025-02-21

**Authors:** Ambarin Faizi, Barbara E. McDermott, Katherine Warburton

**Affiliations:** 1 California Department of State Hospitals; 2 Department of Psychiatry and Behavioral Sciences, University of California, Davis, Sacramento

**Keywords:** forensic psychiatry, psychopharmacology, criminal legal system, public psychiatry, public policy, deinstitutionalization, competency to stand trial

## Abstract

This study explores the effectiveness of antipsychotic medications in restoring competency to stand trial in individuals with severe mental illness, particularly psychotic disorders. While antipsychotic medications are known for reducing symptoms of psychosis, this research focuses on their ability to improve functional outcomes necessary for competency to stand trial (CST). Among over 3,000 patients in California’s forensic state hospital system, 86.5% were successfully restored to competency, with 98.8% discharged on antipsychotic medications. Patients on antipsychotic monotherapy demonstrated higher restoration rates compared to those requiring additional mood stabilizers, suggesting that more complex cases demand more intensive treatment. Delusional disorder, traditionally seen as more resistant to treatment, showed a high restoration rate of 93.8% with antipsychotic use.

Our findings emphasize the pivotal role of antipsychotics in not only reducing symptoms but also in restoring critical functional abilities for participation in legal proceedings. The functional improvements they enable extend beyond the courtroom. Incorporation of antipsychotic medication as an integral evidence-based mechanism in facilitating community reintegration for individuals with severe mental illness supports the broader goal of transitioning individuals from the legal system back into society, consistent with the ultimate promise of deinstitutionalization.

The advancement of antipsychotic medications represents a significant leap in our ability to treat severe mental illness, thus ensuring that individuals can be treated effectively and humanely within the legal system.

—Justice Sandra Day O’Connor, *Riggins v. Nevada* (1992)

## Introduction

The evolution of treatments in psychiatry has significantly transformed over the past century, moving from rudimentary and often inhumane practices to sophisticated pharmacological interventions. The serendipitous discovery of chlorpromazine as a sedative by Henri Laborit, a French surgeon, led to its trial in psychiatric patients due to its observed calming effect. Chlorpromazine demonstrated remarkable efficacy in reducing psychotic symptoms in psychiatric patients, leading to its widespread adoption in psychiatric hospitals.^1^ The discovery of chlorpromazine marked a turning point, as it was the first antipsychotic medication that effectively managed symptoms of psychosis and laid the groundwork for the development of numerous other antipsychotic medications.^1^

The literature contains robust evidence establishing the use of antipsychotic medication in the treatment of schizophrenia and other psychotic disorders primarily via a reduction in the positive symptoms of psychosis (delusions, hallucinations).^2^ ^–^^5^ The landmark, Clinical Antipsychotic Trials of Intervention Effectiveness (CATIE)^6^ study, for instance, compared multiple antipsychotic medications, including both first-generation (typical) and second-generation (atypical) antipsychotics. This large-scale, multiphase study found that while all tested medications were effective in managing psychotic symptoms, differences in side effect profiles significantly influenced patient adherence and outcomes. Multiple studies have demonstrated similar results (CUtLASS, SOHO).^7^ ^–^^8^ Along these lines, a meta-analysis conducted by Leucht and colleagues^9^ provided evidence that long-acting injectable antipsychotics (LAIs) were more effective in preventing relapse compared to their oral counterparts. This study reviewed data from multiple randomized trials and concluded that injectable formulations provided more consistent drug levels, which helped maintain symptom control and reduce relapse rates, thereby offering a valuation option for patients with adherence issues. Several studies support the advantages of long-acting injectables in relapse prevention and reducing hospitalization.^10^ ^–^^12^

The development of these above-described medications with demonstrated efficacy in treating the positive psychotic symptoms of major mental disorders spurred the deinstitutionalization movement of the 1960s and 70s, which sought to reduce the population of mental health patients in institutional settings by shifting the treatment to community-based environments. Unfortunately, this deinstitutionalization movement also led to unintended consequences, such as transinstitutionalization and criminalization of mental illness.^13^ Incarceration rates are notably high among individuals with mental illness, particularly those with psychotic disorders such as schizophrenia.^14^ Approximately 24% of local jail inmates in the United States have reported symptoms of a psychotic disorder, such as delusions or hallucinations, according to the Bureau of Justice Statistics report from 2006.^15^ Although this statistic has remained stable over time, recent reports continue to highlight a significant presence of psychotic disorders among the incarcerated population. The overrepresentation of people with psychotic illness in the legal system is underscored by the ballooning referrals for competency to stand trial (CST) evaluations over the past decade. Estimates of the annual number of competence evaluations have previously ranged from 19,000 to 60,000, but more recent estimates suggest that approximately 94,000 evaluations are conducted annually in the United States.^16^

CST is a fundamental concept in forensic psychiatry and legal proceedings, ensuring that defendants possess the mental capacity to understand the nature and consequences of the legal process and participate adequately in their defense. The landmark case Dusky v. United States (1960) established the standard for competency, requiring that defendants have a rational and factual understanding of the proceedings against them and be able to consult with their attorney with a reasonable degree of rational understanding.^17^ Meta-analyses by Nicholson and Kugler^18^and Pirelli and Zapf^19^ revealed several indicators related to the finding of incompetence to stand trial, notably that individuals diagnosed with a psychotic disorder were eight times more likely to be deemed incompetent than individuals without a psychotic disorder. Active psychosis, including delusions and hallucinations, can distort an individual’s perception of reality, making it challenging for them to engage meaningfully in the legal process.^20^

Antipsychotic medications play a vital role in effectively treating symptoms of psychosis but what impact do they have on improving functional abilities to enable individuals to understand the legal process and participate in their defense? While the efficacy of antipsychotic medications in symptom reduction is well established, their success in restoring the functional competencies necessary for CST is less recognized. Wall and Lee^21^ emphasize that competency encompasses more than just an absence of psychotic symptoms; it involves a complex interplay of cognitive, emotional, and behavioral capacities. Mossman^22^ conducted a comprehensive study that identified antipsychotic medications as essential in restoring competency in defendants diagnosed with schizophrenia and other psychotic disorders. His research demonstrated that with appropriate pharmacological treatment, a significant proportion of incompetent defendants could be restored to competency. His findings were further supported by Cochrane and colleagues^23^ who reviewed the impact of psychotropic medications on trial competency, emphasizing the role of antipsychotics in alleviating symptoms that impede legal competence. Their review highlighted that effective treatment of psychotic symptoms was crucial for restoring cognitive and functional abilities necessary for trial competency. They describe the consistently high response rate to antipsychotic medication treatment as “remarkable.”

Our study’s focus on functional outcomes is predicated on the understanding that competency restoration involves a comprehensive recovery of abilities. This includes the capacity to comprehend the charges against oneself, the roles of various courtroom personnel, and the potential consequences of legal decisions. The Dusky v. United States (1960) ruling emphasizes the necessity for defendants to have both a rational and factual understanding of legal proceedings, which inherently involves higher-level cognitive and functional capabilities. By focusing on these outcomes, our study substantiates the effectiveness of antipsychotic medications in not only reducing symptoms but also improving the functional abilities required for legal competency.

The main purpose of our research was to examine the effectiveness of antipsychotic medication on the real-world functional outcomes of competency restoration. Traditional measures of antipsychotic efficacy primarily focus on the reduction of positive psychotic symptoms such as hallucinations and delusions. While these are crucial for acute symptom management, they do not necessarily translate into the practical abilities required for a defendant to be deemed competent to stand trial. CST requires more than symptom reduction; it necessitates higher-level cognitive and functional abilities to understand court proceedings, communicate effectively with legal counsel, and participate meaningfully in one’s defense.^19^ ^,^^21^ This study examines over 3,000 individuals in California’s state hospital system who were deemed incompetent to stand trial (IST) to determine if antipsychotic medications are not just effective in symptom relief but also have utility in restoring complex functional capabilities.

## Methods

This research was approved by the State of California Committee for the Protection of Human Subjects and the University of California, Davis School of Medicine Institutional Review Board (IRB). The IRB granted a waiver of informed consent.

The study was conducted at the Department of State Hospital (DSH) hospitals using data collected from the records of individuals found IST and committed to DSH for restoration. Of the five existing hospitals in the DSH system, only four provide competency restoration treatment. In these four DSH hospitals, over 85% of the beds are dedicated to patients under forensic commitments, comprised primarily of patients found incompetent to stand trial (IST), Not Guilty by Reason of Insanity (NGRI), and Offenders with Mental Disorders (OMD; a California statute that civilly commits offenders as dangerous after a determinate sentence), with a small number of other commitment types. Although DSH also funds numerous jail-based competence restoration programs in CA, only hospital admissions were examined in this study.

The records of patients found IST and admitted for restoration to competence between the dates of February 1, 2018 through January 31, 2019 were included in the study. At the time of data collection, California statute required that, with few exceptions, defendants found IST for a felony be committed to a locked facility for restoration to competence. Only offenders found IST for misdemeanors and a limited number of non-violent felonies were by statute eligible for community restoration. The maximum length of commitment for restoration of offenders with felony charges was 3 years at the time the majority of this study was conducted. On January 1, 2019, the maximum length of commitment was reduced to 2 years.

### Procedure

All patients were admitted directly from referring county jails. When committed to DSH, courts are required to submit documentation for each individual, which includes documentation of the committing offense and the arrest report associated with the committing offense, the report(s) written by the forensic evaluator(s), the criminal arrest history, any jail medical records available at the time of commitment, and various court documents associated with the commitment (primarily minute orders). A document capturing all relevant information was developed and coders were trained in extracting necessary information for the records. All coders were required to evidence consistency in coding data during this training.

For discharge data, the electronic data systems maintained by DSH were accessed to obtain discharge date, discharge commitment type (primarily restored to competence vs no substantial likelihood of restoration [NSL]), and discharge diagnoses. When restoration status seemed implausible based on the length of hospitalization, the associated court report for that admission was accessed to confirm the evaluator’s opinion. All four hospitals provided access to court reports to confirm discharge recommendations; all reports were examined when the length of hospitalization was one year or longer. When inconsistencies were found, the electronic system indicated restoration whereas the report recommended that the individual be returned to court as NSL. In no case did the electronic data system indicate NSL and the report recommended restoration. Note that the outcome measure in this study was the restoration opinion contained in the report. The judicial decision regarding this opinion was not obtained.

For diagnosis, we accessed the electronic data management system that tracks treatment team diagnosis. For this study, we opted to use discharge diagnosis, as admission diagnosis is often unreliable due to the treatment team’s unfamiliarity with patients on admission. Moreover, because the electronic data management system tracks treatment team diagnosis, which may not align with the diagnosis contained in the court report, we opted to only access the electronic system.

To determine the types of medications used for restoration, we accessed the electronic data system documenting medications. This system maintains records of all medications prescribed during the individual’s hospitalization. Because of the complexity of prescribing practices in long-term care facilities and the types of individuals found not competent to stand trial (typically diagnosed with either a psychotic disorder or a cognitive disorder), we examined the data to document the use of two classes of medications: antipsychotics (either typical or atypical) and mood stabilizers. Dosage was not recorded for these analyses. Each individual was reported as either prescribed an antipsychotic (yes/no) or a mood stabilizer (yes/no). Each patient was categorized as discharged on neither an antipsychotic nor a mood stabilizer, only an antipsychotic, only a mood stabilizer, or on both an antipsychotic and a mood stabilizer.

### Participants

There were a total of 3166 unduplicated IST admissions during the specified time period. Twenty-two individuals were recommitted during the year this study was conducted, only one of whom was committed as IST for a new offense. The remainder were recommitted for the same offense after their initial restoration. For these 22 individuals, only their first admission was retained in the dataset.

As can be seen in Table 1, women comprised 17.6% of the admissions (n = 556), with the remainder (n = 2610, 82.4%) described as male. Approximately one-third of the admissions were White (n = 1046, 33.1%), with slightly less than one-third Hispanic (n=994, 31.4%). The remainder were either Black (n=896, 28.3%) or of another ethnic background, including American Indian and Asian (n = 226, 7.1%). The age of the patients on admission ranged from 18 to 92, with an average age of 38.6 (std = 12.6). The majority of patients had documentation of at least one prior inpatient psychiatric admission as an adult (n = 1756, 56.5%), although many records did not indicate any prior psychiatric history, either inpatient or outpatient (n = 802, 25.8%). The remainder had documentation that reflected a history of mental health treatment only as a juvenile, as an outpatient, or receiving psychiatric medications from a non-mental health provider (n = 551, 17.7%). Most admissions were English speaking (n = 2956, 94.7%), although 127 were documented as speaking Spanish as their primary language (4.1%). A very small percentage had records that reflected speaking another language (n = 36, 1.2%). Only the most serious felony offense for which the person was found IST was recorded as the commitment offense. The most common commitment offense was assault/battery (n = 1157, 37.1%), followed by theft (n = 404, 12.9%), robbery (n = 254, 8.1%), criminal threats (n = 253, 8.1%), miscellaneous charges (typically vandalism, n = 172, 5.5%), obstruction of justice (171, 5.5), homicide offenses (164, 5.3), arson (n=147,4.7%), failure to register as a sex offender (n = 52, 1.7%), and other (kidnapping, white collar crimes, major driving offenses, escape, (n = 75, 2.4%)).

**Table 1.** Demographic and clinical characteristics of the sample

	n (%)
Gender	
Female	556 (17.6)
Male	2610 (82.4)
Race	
African-American	896 (28.3)
Caucasian	1046 (33.1)
Hispanic	994 (31.4)
Other Non-white	226 (7.1)
Prior psychiatric treatment	
Inpatient (as an adult)	1756 (56.5)
None	802 (25.8)
Only juvenile/outpatient/nonpsychiatric provider	551 (17.7)
Commitment offense	
Assault/battery	1157 (37.1)
Theft	404 (12.9)
Robbery	254 (8.1)
Criminal threats	253 (8.1)
Miscellaneous	172 (5.5)
Obstruction of justice	171 (5.5)
Homicide offenses	164 (5.3)
Arson	147 (4.7)
Failure to register (sex offense)	52 (1.7)
Other	75 (2.4)

### Data analysis

Data were analyzed using SPSS version 29. Statistical analyses included frequency distributions and descriptive statistics to provide information regarding basic demographics. Chi-square and mean differences analyses were conducted to assess differences in categorical and continuous variables respectively.

## Results

Of the 3166 individuals admitted during the specified time period, 2733 (86.5%) were discharged as restored to competence and 392 (12.5%) were discharged as NSL. The remaining 41 were not discharged and were either returned to court as competent but remained in the hospital to maintain competence (n = 18, 0.6%) or remained in the hospital under a different commitment (for example, found NGRI and hospitalization continued under that commitment, n = 23, 0.7%).

Our primary hypothesis was that antipsychotic medication was critical and effective in restoring patients found not competent to stand trial. As can be seen from Table 2, fully 98.8% of individuals admitted as IST were discharged on an antipsychotic, with the majority discharged on an antipsychotic alone (70.9%) and an additional 27.9% on both an antipsychotic and a mood stabilizer. This table also demonstrates that almost 90% of individuals discharged on only an antipsychotic were believed to be restored to competence, in comparison to patients discharged on a combination of an antipsychotic and a mood stabilizer, where only slightly more than 80% of these individuals were believed restored. When patients were discharged on neither an antipsychotic nor a mood stabilizer, close to 95% were restored to competence. The two patients returned to court as NSL and on neither of the targeted medications had a primary diagnosis of a neurocognitive disorder. All patients discharged on only a mood stabilizer were restored to competence (X^2^(3) = 38.5, p < .001). This table clearly shows that individuals for whom an antipsychotic was not sufficient were less likely to be restored than any other group.

**Table 2.** Restoration status by discharge medication

	Neither antipsychotic nor mood stabilizer	Only antipsychotic	Antipsychotic plus mood stabilizer	Only mood stabilizer
	N (%)	N (%)	N (%)	
Restored	34 (94.4)	1984 (89.6)	711 (81.6)	4 (100)
Returned as no substantial likelihood	2^a^ (5.6)	230 (10.4)	160 (18.4)	0 (0)
Total	36 (1.1)	2214 (70.9)	871 (27.9)	4 (0.1)

a DC diagnosis of a neurocognitive disorder

Since psychiatric medications may not be the treatment of choice for certain disorders (for example neurocognitive disorders are unlikely to respond to antipsychotic medications), we examined the discharge diagnoses associated with medications and restoration status. Tables 3–5 clarify the relationship between diagnosis, medications, and discharge status. As can be seen from Table 3, the plurality of patients received a diagnosis of schizophrenia; the second largest diagnostic category was unspecified schizophrenia spectrum and other psychotic disorders, followed by schizoaffective disorder. Combined with delusional disorder, these four diagnostic categories, whose primary symptoms are psychotic in nature, comprised over 75% of the total sample. Notably, three diagnoses evidenced restoration rates of less than 90%: schizophrenia, schizoaffective disorder, and neurocognitive disorders. Not surprisingly, neurocognitive disorders had substantially lower restoration rates than any other diagnosis (less than 40% were restored to competence) although they comprised a very small percentage of the total sample.

**Table 3.** Restoration to competence by discharge diagnoses

Discharge diagnosis	Total sample with discharge diagnosis, outcome, and medication	Percent discharged as restored to competence	Percent discharged as no substantial likelihood
	N (%)	N (%)	N (%)
Schizophrenia	1169 (38.0)	987 (84.4)	182 (15.6)
Schizoaffective disorder	483 (15.7)	420 (87.0)	63 (13.0)
Bipolar disorder	261 (8.5)	252 (96.6)	9 (3.4)
Unspecified schizophrenia spectrum and other psychotic disorders	703 (22.8)	655 (93.2)	48 (6.8)
Neurocognitive disorders	73 (2.4)	29 (39.7)	44 (60.3)
Delusional disorder	32 (1.0)	30 (93.8)	2 (6.3)
Other mental health disorders	132 (4.3)	120 (90.9)	12 (9.1)
No major mental health disorder	226 (7.3)	220 (97.3)	6 (2.7)
Total	3079	2713 (88.1)	366 (11.9)

Table 4 provides restoration rates and the percentage of the total sample who were returned to court prescribed only an antipsychotic. Not surprisingly, two diagnostic categories evidenced a lower percentage of patients returned to court—as either restored or NSL—on only an antipsychotic. Less than 50% of individuals with a diagnosis of schizoaffective disorder were returned to court only prescribed an antipsychotic; slightly more than 60% of individuals with a diagnosis of bipolar disorder were discharged prescribed only an antipsychotic.

**Table 4.** Restoration to competence by discharge diagnosis—discharged only on an antipsychotic

Discharge diagnosis	Percent of sample discharged on antipsychotic alone	Percent discharged as restored to competence	Percent discharged as no substantial likelihood
	N (% of total sample)	N (%)	N (%)
Schizophrenia	880 (75.5)	766 (87.0)	114 (13.0)
Schizoaffective disorder	239 (49.8)	218 (91.2)	21 (8.8)
Bipolar disorder	158 (60.8)	153 (96.8)	5 (3.2)
Unspecified schizophrenia spectrum and other psychotic disorders	563 (80.7)	530 (94.1)	33 (5.9)
Neurocognitive disorders	52 (76.5)	20 (38.5)	32 (61.5)
Delusional disorder	26 (83.9)	25 (96.2)	1 (3.8)
Other mental health disorders	93 (75.0)	85 (91.4)	8 (8.6)
No major mental health disorder	180 (84.9)	176 (97.8)	4 (2.2)
Total	2191 (72.1)	1973 (90.1)	218 (9.9)

Table 5 documents the diagnostic comparisons for restoration rates in individuals who required the addition of a mood stabilizer to their medication regimen. As can be seen in this table, substantially more people who required adjunctive medication were unlikely to be restored for most diagnoses with two notable exceptions: bipolar disorder and neurocognitive disorders. Individuals with these diagnoses evidenced a similar restoration rate with or without the addition of a mood stabilizer (96.8 and 96.1 for bipolar disorder and 38.5 and 37.5 for neurocognitive disorders).

**Table 5.** Restoration to competence by discharge diagnosis – discharged on antipsychotic and mood stabilizer

Discharge diagnosis	Percent of sample discharged on both antipsychotic and mood stabilizer	Percent discharged as restored to competence	Percent discharged as No Substantial Likelihood
	N (% of total sample)	N (%)	N (%)
Schizophrenia	286 (24.5)	218 (76.2)	68 (23.8)
Schizoaffective disorder	241 (50.2)	199 (82.6)	42 (17.4)
Bipolar disorder	102 (39.2)	98 (96.1)	4 (3.9)
Unspecified schizophrenia spectrum and other psychotic disorders	135 (19.3)	120 (88.9)	15 (11.1)
Neurocognitive disorders	16 (23.5)	6 (37.5)	10 (62.5)
Delusional disorder	5 (16.1)	4 (80.0)	1 (20.0)
Other mental health disorders	31 (25.0)	27 (87.1)	4 (12.9)
No major mental health disorder	32 (15.1)	30 (93.8)	2 (6.2)
Total	848 (27.9)	702 (82.8)	146 (17.2)

Table 6 provides the length of stay by discharge diagnosis for patients who were returned to court as restored to competence. Not surprisingly, individuals diagnosed with neurocognitive disorders required the longest hospitalization (average of almost 6 months), followed by individuals diagnosed with schizophrenia (slightly less than 5 months). Patients diagnosed with schizoaffective disorder also differed from all other diagnostic categories with a discharge length of stay of slightly more than 4 months. All other diagnostic categories evidenced lengths of stay of approximately 3.5 months or less. The shortest lengths of stay were found in individuals with either no major mental health disorder (i.e., personality disorders and substance use disorders) or nonpsychotic mental health disorders (e.g., anxiety disorders). Interestingly, individuals with a diagnosis of a delusional disorder evidenced a relatively brief length of stay of approximately 3 months.

**Table 6.** Days in hospital by diagnosis—discharged restored

Discharge diagnosis	Days in hospital
	Mean (Std)
Schizophrenia^b^	145.0 (121.0)
Schizoaffective disorder^c^	130.5 (103.7)
Bipolar disorder^d^	107.0 (86.1)
Unspecified schizophrenia spectrum and other psychotic disorder^d^	107.5 (94.2)
Neurocognitive disorders^a^	179.7 (109.4)
Delusional disorder^d^	97.4 (67.1)
Other mental health disorders^d^	78.4 (62.0)
No major mental health disorder	77.4 (74.9)

a Neurocognitive disorders longer than all others

b Schizophrenia different than all others

c Schizoaffective different than all others

d All statistically same

**Figure 1.**
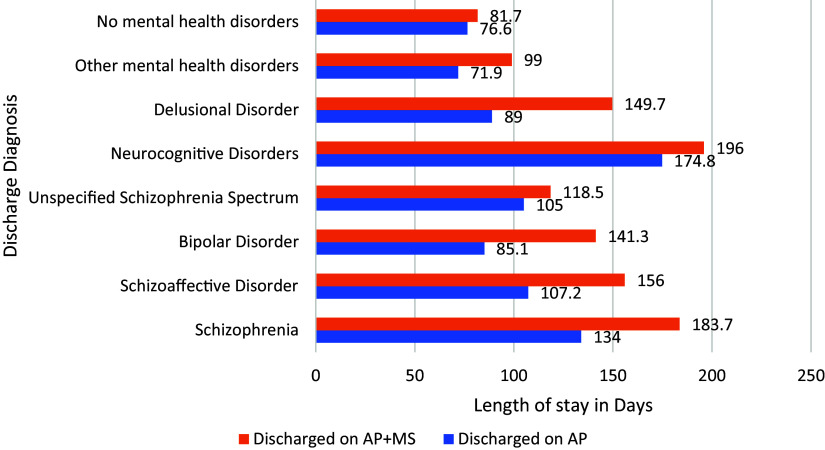
Days in hospital by diagnosis and medication—discharge restored.

Figure 1 depicts the restoration discharge length of stay by diagnosis for individuals restored on an antipsychotic alone compared to those requiring the addition of a mood stabilizer. As can be seen from this graphic, individuals requiring the addition of a mood stabilizer evidenced longer discharge lengths of stay in general (F(7,2659) = 2.012, p = .05). However, for three diagnostic categories (unspecified schizophrenia spectrum and other psychotic disorders, neurocognitive disorders, and no major mental health disorders) the differences were not statistically significant.

## Discussion

Our data provide compelling evidence that antipsychotic medications are critical in restoring competency to proceed with criminal charges. Our primary question was, “Do antipsychotic medications work?,” with an operational definition of “work” entirely different than most research on these medications. We were not attempting to understand if antipsychotic medication reduced psychotic symptoms. As noted previously, there is ample evidence that in the majority of individuals diagnosed with a psychotic disorder, both first-generation (typical) and second-generation (atypical) antipsychotic medications reduce the positive symptoms of psychosis.^2^ ^–^^8^ Moreover, there is some research that suggests that certain atypical medications reduce cognitive or negative symptoms observed in some psychotic disorders.^4^ ^–^^5^ But is that definition of “work” enough? A recent New York Times opinion essay^24^ argued that merely decreasing or eliminating symptoms was an inadequate definition of work. The author noted that while approximately 60% of individuals with a psychotic disorder experience symptom remission or reduction, some studies describe worsening outcomes with long-term maintenance of these medications, noting “profound side effects” associated with these medications. The author argued that because of these negative outcomes, antipsychotic medications should not be a mandatory component of treatment and that alternative interventions should be explored. Admittedly, despite their efficacy in managing the positive symptoms of schizophrenia, medications such as chlorpromazine (known as first generation or typical antipsychotics) are associated with adverse events such as extrapyramidal symptoms (EPS) like tardive dyskinesia.^25^ The introduction of clozapine in the 1970s marked the beginning of the second-generation antipsychotics, also known as atypical antipsychotics. These medications aimed to balance efficacy and adverse reactions, although they introduced other challenges such as metabolic side effects, including weight gain and diabetes.^26^ Recent advancements in antipsychotic therapy have focused on optimizing efficacy, minimizing side effects, and improving patient adherence. LAIs have been developed to enhance treatment adherence by providing sustained drug release, reducing the need for daily medication.^27^ These formulations have demonstrated efficacy in preventing relapse and improving outcomes in schizophrenia.^28^ Even more recently, in September 2024 the FDA approved an antipsychotic medication that employs a novel mechanism of action for reducing psychotic symptoms, which may further reduce negative side effects.^29^ This reduction in side effects may increase adherence and improve long-term outcomes.

Our study’s findings underscore the pivotal role of antipsychotic medications in restoring functional capacity via the restoration of CST among individuals diagnosed with severe mental illness, particularly psychotic disorders. The efficacy of antipsychotics in reducing symptoms of psychosis is well-established, yet our research highlights their critical role in restoring the functional capabilities necessary for CST. Among the 3,166 individuals admitted as IST, 86.5% were successfully restored to competency, with 98.8% of these individuals discharged on an antipsychotic regimen, highlighting the crucial role these medications play in not only symptom reduction but also in functional restoration.

One of the key findings of our study is the differential outcomes observed among individuals prescribed a combination of antipsychotics and mood stabilizers versus those on antipsychotic monotherapy. Patients requiring the addition of mood stabilizers demonstrated longer lengths of stay and higher rates of being deemed as having no substantial likelihood of restoration to competency, indicating a more complex and treatment-resistant illness. This finding is consistent with an international study^30^ in 2020 which demonstrates that patients with schizophrenia who received adjunctive treatment with mood stabilizers are more severely ill and less responsive to monotherapy treatments. In our study specifically, while 89.6% of individuals discharged on antipsychotic monotherapy were restored to competency, this rate decreased to 81.6% for those on both an antipsychotic and a mood stabilizer. These findings suggest that the presence of mood instability or comorbid conditions, such as schizoaffective or bipolar disorder, contributes to the complexity of psychotic illness, necessitating more intensive and prolonged treatment efforts.

Our study also sheds light on the treatability of delusional disorder with antipsychotic medications, a disorder traditionally viewed as more resistant to treatment than other psychotic disorders. Notably, our data indicate that individuals diagnosed with the delusional disorder who were treated with antipsychotics had a restoration rate of 93.8% and a relatively brief length of stay of approximately 3 months, compared to an average of 5 months for patients with schizophrenia. A critical aspect of this more rapid response to medication may lie in the symptom profile of these two diagnostic categories. The literature documents a wide range of symptoms associated with a schizophrenic process, which can include the previously described positive symptoms as well as negative symptoms, including various deficits in cognitive functioning. In contrast, most literature describing symptoms associated with delusional disorder documents that cognitive function remains intact regardless of medication intervention.^31^ ^–^^33^ This diagnostic difference in symptom profiles may lead to a more rapid restoration rate. Regardless, our data supports emerging literature^34^ ^–^^36^ that suggests delusional disorder can indeed be effectively managed with antipsychotic medication, leading to significant functional improvement and restoration of competency.

A key strength of our study is its large sample size of 3,166 individuals, which enhances the generalizability of our findings across diverse treatment settings. Additionally, our focus on functional outcomes provides a more comprehensive understanding of the effectiveness of antipsychotic medications beyond mere symptom reduction.

However, our study is not without limitations. The reliance on discharge diagnoses may introduce variability in diagnostic accuracy, and the absence of dosage data limits our ability to assess the impact of specific medication regimens. Future research should explore the role of dosage and the potential benefits of combining pharmacological and non-pharmacological interventions to optimize functional outcomes.

## Conclusions

The use of antipsychotic medication has been validated by extensive clinical research demonstrating its efficacy in managing symptoms of several mental disorders such as schizophrenia and bipolar disorder. Antipsychotics reduce delusions, hallucinations, and other psychotic features, allowing patients to function more effectively in daily life. While critics may argue that compulsory mental health care overemphasizes medication, it is crucial to note that antipsychotics, when appropriately administered and managed, can significantly improve patients’ quality of life, prevent relapse, and support long-term recovery and stability.

In conclusion, our study affirms the vital role of antipsychotic medications in restoring competency among individuals with severe mental illness, particularly those with psychotic disorders. The effectiveness of antipsychotic medications, including in the treatment of delusional disorder and comorbid mood symptoms, provides a hopeful outlook for restoring functionality and increasing independence in a vulnerable patient population. While the introduction of antipsychotic medication catalyzed the deinstitutionalization movement and was heralded as a shift toward greater societal integration for individuals with mental illness, the current state of individuals with severe mental illness and criminal legal system involvement reflects a significant divergence from that vision. Rather than being fully realized as an evidence-based treatment mechanism for facilitating meaningful community reintegration, medications are often used primarily to stabilize individuals for participation in the criminal legal system. This highlights an unfortunate systemic shortfall in achieving the intended promise of community-based care.

## References

[1] Delay J, Deniker P, Harl JM. Therapeutic use in psychiatry of phenothiazine of central elective action. Ann Med Psychol (Paris). 1952 Jun;110(2 1):112–117. PMID: 12986408. 12986408

[2] Leucht S, Tardy M, Komossa K, Heres S, Kissling W, Salanti G, Davis JM. Antipsychotic drugs versus placebo for relapse prevention in schizophrenia: a systematic review and meta-analysis. Lancet. 2012 Jun 2;379(9831):2063–2071. doi: 10.1016/S0140-6736(12)60239-6. 22560607

[3] Howner K, Andiné P, Engberg G, Ekström EH, Lindström E, Nilsson M, Radovic S, Hultcrantz M. Pharmacological treatment in forensic psychiatry-a systematic review. Front Psychiatry. 2020 Jan 16;10:963. doi: 10.3389/fpsyt.2019.00963. 32009993 PMC6976536

[4] Singh A, Delgado D, Ventura MI, Schwartz E, Williams J, Meyer JM. Clozapine use and forensic outcomes in psychiatric inpatients deemed incompetent to stand trial. J Am Acad Psychiatry Law. 2022 Sep;50(3):427–433. doi: 10.29158/JAAPL.210123-21. 35798392

[5] Geddes J, Freemantle N, Harrison P, Bebbington P. Atypical antipsychotics in the treatment of schizophrenia: systematic overview and meta-regression analysis. BMJ. 2000 Dec 2;321(7273):1371–1376. doi: 10.1136/bmj.321.7273.1371. 11099280 PMC27538

[6] Lieberman, JA, Stroup, TS, McEvoy, JP, et al. Effectiveness of antipsychotic drugs in patients with chronic schizophrenia. N Engl J Med. 2005 Sep 22;353(12):1209–1223. doi: 10.1056/NEJMoa051688. 16172203

[7] Jones PB, Barnes TR, Davies L, Dunn G, Lloyd H, Hayhurst KP, Murray RM, Markwick A, Lewis SW. Randomized controlled trial of the effect on Quality of Life of second- vs first-generation antipsychotic drugs in schizophrenia: Cost Utility of the Latest Antipsychotic Drugs in Schizophrenia Study (CUtLASS 1). Arch Gen Psychiatry. 2006 Oct;63(10):1079–1087. doi: 10.1001/archpsyc.63.10.1079. 17015810

[8] Haro JM, Edgell ET, Novick D, Alonso J, Kennedy L, Jones PB, Ratcliffe M, Breier A; SOHO advisory board. Effectiveness of antipsychotic treatment for schizophrenia: 6-month results of the Pan-European Schizophrenia Outpatient Health Outcomes (SOHO) study. Acta Psychiatr Scand. 2005 Mar;111(3):220–231. doi: 10.1111/j.1600-0447.2004.00450.x. 15701107

[9] Leucht C, Heres S, Kane JM, Kissling W, Davis JM, Leucht S. Oral versus depot antipsychotic drugs for schizophrenia – A critical systematic review and meta-analysis of randomized long-term trials. Schizophr Res. 2011 Apr;127(1–3):83–92. doi: 10.1016/j.schres.2010.11.020. 21257294

[10] Tiihonen J, Wahlbeck K, Lönnqvist J, Klaukka T, Ioannidis JP, Volavka J, Haukka J. Effectiveness of antipsychotic treatments in a nationwide cohort of patients in community care after first hospitalisation due to schizophrenia and schizoaffective disorder: observational follow-up study. BMJ. 2006 Jul 29;333(7561):224. doi: 10.1136/bmj.38881.382755.2F. 16825203 PMC1523484

[11] Tiihonen J, Haukka J, Taylor M, Haddad PM, Patel MX, Korhonen P. A nationwide cohort study of oral and depot antipsychotics after first hospitalization for schizophrenia. Am J Psychiatry. 2011 Jun;168(6):603–609. doi: 10.1176/appi.ajp.2011.10081224. 21362741

[12] Lambert T, Olivares JM, Peuskens J, DeSouza C, Kozma CM, Otten P, Crivera C, Jacobs A, Macfadden W, Mao L, Rodriguez SC, Dirani R, Akhras KS. Effectiveness of injectable risperidone long-acting therapy for schizophrenia: data from the US, Spain Australia, and Belgium. Ann Gen Psychiatry. 2011 Apr 4;10:10. doi: 10.1186/1744-859X-10-10. 21463526 PMC3090384

[13] Hoge SK. Providing transition and outpatient services to the mentally ill released from correctional institutions. In: Greifinger RB, ed. Public Health Behind Bars. New York: Springer; 2022:445–460. 10.1007/978-1-0716-1807-3_30.

[14] Ramsay CE, Goulding SM, Broussard B, Cristofaro SL, Abedi GR, Compton MT. Prevalence and psychosocial correlates of prior incarcerations in an urban, predominantly African-American sample of hospitalized patients with first-episode psychosis. J Am Acad Psychiatry Law. 2011;39(1):57–64. 21389167 PMC3612963

[15] James DJ, Glaze LE. Mental health problems of prison and jail inmates. Bureau of Justice Statistics Special Report, NCJ 213600. September 2006

[16] Owen EA, Perry A, Scher DP. Trauma in competency to stand trial evaluations. In: Javier RA, Owen EA, Maddux JA, eds. Assessing Trauma in Forensic Contexts. Switzerland AG: Springer Nature; 2020: 65–84. 10.1007/978-3-030-33106-1_3

[17] Dusky V. United States, 362 U.S. 402. 1960

[18] Nicholson RA, Kugler KE. Competent and incompetent criminal defendants: a quantitative review of comparative research. Psychol Bull. 1991 May;109(3):355–370. doi: 10.1037/0033-2909.109.3.355. 2062978

[19] Pirelli G, Gottdiener WH, Zapf PA. A meta-analytic review of competency to stand trial research. Psychol Pub Pol’y Law. 2011; 17(1): 1–53. 10.1037/a0021713

[20] Rosenfeld B, Wall BW. Psychopathology and competence to stand trial. Crim Justice Behav. 1998; 25(4):443–462. 10.1037/a0021713

[21] Wall B, Lee R. Assessing competency to stand trial. Psychiatr Times. 2020;37(10).

[22] Mossman D. Predicting restorability of incompetent criminal defendants. J Am Acad Psychiatry Law. 2007;35(1):34–43. 17389343

[23] Cochrane RE, Herbel BL, Reardon ML, Lloyd KP. The Sell effect: Involuntary medication treatment is a “clear and convincing” success. Law Hum Behav. 2013;37(2): 107–116. 10.1037/lhb0000003 22746284

[24] Bergner D. A major problem with compulsory mental health care is the medication. The New York Times. 2023.

[25] Casey DE. Pathophysiology of antipsychotic drug-induced movement disorders. J Clin Psychiatry. 2004;65(Suppl 9):25–28. 15189109

[26] Newcomer JW. Second-generation (atypical) antipsychotics and metabolic effects: a comprehensive literature review. CNS Drugs. 2005;19(S uppl 1):1–93. doi: 10.2165/00023210-200519001-00001. 15998156

[27] Fleischhacker WW, Meise U, Günther V, Kurz M. Compliance with antipsychotic drug treatment: influence of side effects. Acta Psychiatr Scand Suppl. 1994;382:11–15. 7916523

[28] Kishimoto T, Hagi K, Kurokawa S, Kane JM, Correll CU. Long-acting injectable versus oral antipsychotics for the maintenance treatment of schizophrenia: a systematic review and comparative meta-analysis of randomised, cohort, and pre-post studies. Lancet Psychiatry. 2021 May;8(5):387–404. doi: 10.1016/S2215-0366(21)00039-0. 33862018

[29] Meyers JM. How antipsychotics work in schizophrenia: A primer on mechanisms. *CNS Spectrums*, In Press. 10.1017/S1092852924002244 PMC13064704 39618418

[30] Lim WK, Chew QH, He YL, et al. Coprescription of mood stabilizers in schizophrenia, dosing, and clinical correlates: An international study. Hum Psychopharmacol. 2020; 35: 1–7. doi: 10.1002/hup.2752. 32738085

[31] Lähteenvuo M, Taipale H, Tanskanen A, Mittendorfer-Rutz E, Tiihonen J. Effectiveness of pharmacotherapies for delusional disorder in a Swedish national cohort of 9076 patients. Schizophr Res. 2021 Feb;228:367–372. doi: 10.1016/j.schres.2021.01.015. 33548837

[32] González-Rodríguez A, Seeman MV. Differences between delusional disorder and schizophrenia: a mini narrative review. World J Psychiatry. 2022 May 19;12(5):683–692. doi: 10.5498/wjp.v12.i5.683. 35663297 PMC9150033

[33] Muñoz-Negro JE, Ibáñez-Casas I, de Portugal E, Lozano-Gutiérrez V, Martínez-Leal R, Cervilla JA. A Psychopathological Comparison between Delusional Disorder and Schizophrenia. Can J Psychiatry. 2018 Jan;63(1):12–19. doi: 10.1177/0706743717706347. 28595494 PMC5788118

[34] Manschreck TC, Khan NL. Recent advances in the treatment of delusional disorder. Can J Psychiatry. 2006 Feb;51(2):114–119. doi: 10.1177/070674370605100207. 16989110

[35] Miola A, Salvati B, Sambataro F, Toffanin T. Aripiprazole for the treatment of delusional disorders: a systematic review. Gen Hosp Psychiatry. 2020 Sep-Oct;66:34–43. doi: 10.1016/j.genhosppsych.2020.06.012. 32650190

[36] Gunduz-Bruce H, McMeniman M, Robinson DG, Woerner MG, Kane JM, Schooler NR, Lieberman JA. Duration of untreated psychosis and time to treatment response for delusions and hallucinations. Am J Psychiatry. 2005 Oct;162(10):1966–1969. doi: 10.1176/appi.ajp.162.10.1966. 16199847

